# Fermentation products in the cystic fibrosis airways induce aggregation and dormancy-associated expression profiles in a CF clinical isolate of *Pseudomonas aeruginosa*

**DOI:** 10.1093/femsle/fny082

**Published:** 2018-03-29

**Authors:** Joann Phan, Tara Gallagher, Andrew Oliver, Whitney E England, Katrine Whiteson

**Affiliations:** Department of Molecular Biology and Biochemistry, University of California at Irvine, 3315 McGaugh Hall, UCI, Irvine, CA 92697, USA

**Keywords:** fermentation products, transcriptomics, metabolomics, dormancy, aggregation, *Pseudomonas aeruginosa*

## Abstract

*Pseudomonas aeruginosa* is a well-known dominant opportunistic pathogen in cystic fibrosis (CF) with a wide range of metabolic capacities. However, *P. aeruginosa* does not colonize the airways alone, and benefits from the metabolic products of neighboring cells—especially volatile molecules that can travel between different parts of the airways easily. Here, we present a study that investigates the metabolic, gene expression profiles and phenotypic responses of a *P. aeruginosa* clinical isolate to fermentation products lactic acid and 2,3-butanediol, metabolites that are produced by facultative anaerobic members of the CF polymicrobial community and potential biomarkers of disease progression. Although previous studies have successfully investigated the metabolic and transcriptional profiles of *P. aeruginosa*, most have used common lab reference strains that may differ in important ways from clinical isolates. Using transcriptomics and metabolomics with gas chromatography time of flight mass spectrometry, we observe that fermentation products induce pyocyanin production along with the expression of genes involved in *P. aeruginosa* amino acid utilization, dormancy and aggregative or biofilm modes of growth. These findings have important implications for how interactions within the diverse CF microbial community influence microbial physiology, with potential clinical consequences.

## Teaser

In response to fermentation products 2,3-butanediol and lactic acid, expression patterns associated with aggregation and dormancy are induced in *Pseudomonas aeruginosa*.

## INTRODUCTION

Polymicrobial communities inhabiting the airways of cystic fibrosis (CF) patients carry out diverse metabolisms, producing metabolites that impact both microbial and human cell physiology. Airway infection and inflammation are the primary cause of disease progression in CF. However, basic questions remain about microbial involvement in cystic fibrosis pulmonary exacerbations (CFPE), the acute but difficult to define periods of worsened lung function that lead to irreversible lung damage. In addition to well-known Gram-negative pathogens such as *Pseudomonas aeruginosa*, fermenting bacteria such as *Rothia mucilaginosa* and *Streptococcus* spp. that produce lactic acid and 2,3-butanedione (Whiteson *et al.*^2014^; Phan *et al.*^2017^), also inhabit the airways. We are interested in identifying the physiological response of *P. aeruginosa* to ubiquitous metabolites from the pH neutral fermentation 2,3-butanediol pathway that is distinct to a subset of bacteria along with lactic acid that can be from human or microbial metabolism. We hypothesize that these fermentation products influence *P. aeruginosa* physiology, driving growth rates, expression and metabolic profiles in in clinically relevant ways, reflecting a chronic infection in CF.

Heterogeneous biochemical conditions in the dense mucus layer lining the CF airways consists of steep gradients of pH, oxygen and metabolites that are created by microbial community niches (Worlitzsch *et al.*^2002^; Cowley *et al.*^2015^). Typical microbial residents of the oral cavity including several anaerobes, *Streptococcus* spp. and *R. mucilaginosa* have access to the lung and airways as well, priming the environment for Gram-negative opportunistic pathogens to colonize (Tunney *et al.*^2008^; Kolenbrander 2011; Flynn *et al.*^2016^). In mucin-rich environments such as the CF airway, *Pseudomonas* is unable to catabolize mucins as a carbon source. However, oral microbes efficiently degrade mucins to produce amino acids and short-chain fatty acids that stimulate the growth of *P. aeruginosa* (Flynn *et al.*^2016^). In these cases, interspecies interactions are necessary for survival and colonization.

Interspecies interactions in the CF airway may be driven by microbial metabolites, especially small volatile molecules that can travel throughout the airways easily and result in the altered microbial physiology or increased pathogenicity. One outcome of cross-feeding between fermenters and *P. aeruginosa* is the enhanced production of redox-active and toxic products by *P. aeruginosa*. For example, the pH neutral fermentation product 2,3-butanediol increases *P. aeruginosa* virulence, pyocyanin production, biofilm formation *in vitro* and promotes *P. aeruginosa* lung colonization and inflammation in a mouse model (Venkataraman *et al.*^2014^; Nguyen *et al.*^2016^). Pyocyanin is traditionally thought of as an antagonistic compound used to elicit oxidative stress in competing microbes and host cells. The redox nature of pyocyanin suggests that this molecule may also play a role in primary metabolism as an alternative electron acceptor for *P. aeruginosa* in low-oxygen environments (Glasser, Kern and Newman 2014). As pyocyanin is associated with increased inflammation and worsening function in CF airways (Hunter *et al.*^2012^), metabolites that induce pyocyanin production in *P. aeruginosa* could act as indicators or triggers of CFPE.

Observed increases in levels of 2,3-butanedione and lactic acid during CFPE underscores the importance of understanding the effect of fermentation on the microbial community in the CF airways. Lactic acid concentration has been shown to significantly increase in patients with pre-acute pulmonary exacerbations and significantly decrease after 2–3 weeks of intravenous antibiotics (Bensel *et al.*^2011^; Zang *et al.*^2017^). Twomey *et al.* detected increased levels of lactate in patients experiencing exacerbations compared to stable patients (Twomey *et al.*^2013^). 2,3-butanedione, produced in the same pathway as 2,3-butanediol, is increased in the breath of CF patients compared to healthy individuals (Whiteson *et al.*^2014^). After antibiotic administration for CFPE, 2,3-butanedione decreased in concentration, indicating an association between successful treatment of CFPE and a decrease in 2,3-butanedione (Whiteson *et al.*^2014^).

Many *Pseudomonas* studies use model lab strains such as *P. aeruginosa* PA14 (Turner *et al.*^2015^; Price *et al.*^2016^; Tata *et al.*^2016^; Flynn, Phan and Hunter 2017). However, *P. aeruginosa* strains do not all respond to metabolic signals in the same manner (Leão *et al.*^2010^; Duong *et al.*^2015^; Koehorst *et al.*^2016^). Because lactic acid and 2,3-butanediol are potential biomarkers for CF disease progression, we must better understand the response of *P. aeruginosa* clinical isolates to these metabolites. Here, we investigate how a *P. aeruginosa* CF clinical isolate responds to 2,3-butanediol and lactic acid using multi-omics approaches including transcriptomics and metabolomics in addition to phenotypic assays. We find that in response to 2,3-butanediol and lactic acid, expression patterns associated with aggregative modes of growth and dormancy are induced in *P. aeruginosa*.

## MATERIALS AND METHODS

### Strain and growth conditions

The strain used for this study was PaFLR01, a *Pseudomonas aeruginosa* strain isolated from the sputum of a CF patient. PaFLR01 does not display a mucoidy phenotype or contain the lasR or hypermutator mutations. PaFLR01 was sequenced on an Illumina MiSeq. Sequencing read quality was checked with Fastqc (http://www.bioinformatics.babraham.ac.uk/projects/fastqc/), the genome was assembled with A5 (Tritt *et al.*^2012^), and annotated with Rapid Annotations using Subsystems Technology (RAST) (Aziz *et al.*^2008^). The a5 pipeline combines quality filtering, adapter removal, error checking and scaffold generation and verification (Tritt *et al.*^2012^). The assembly generated 79 scaffolds with a genome size of 6,175,434 base pairs. However, we removed the last eight scaffolds because they were repeats of other larger scaffolds. The genome is available on National Center for Biotechnology Information (NCBI; accession number PXNR00000000, BioSample SAMN08559939, BioProject PRJNA434465) and Patric (https://www.patricbrc.org/workspace/tgallagh@patricbrc.org/Genomes/FLR01; Note: access to data requires a free Patric account login).

PaFLR01 was inoculated into 2 mL Todd Hewitt (TH) broth and grown at 37°C, shaking at 200 rpm. Overnight cultures were diluted with TH broth to OD500 0.05 and transferred to a 96-well plate. For experimental conditions, lactic acid or 2,3-butanediol were added to the cultures at a final concentration of 20 mM. Each condition, including control, was performed in triplicate. The 96-well plate was statically incubated at 37°C for 48 h, well past the exponential growth phase. In TH broth, the *D*-lactate concentration is 1.061 mM and *L*-lactate concentration is 1.241 mM. For the control, the true concentration PaFLR01 was being exposed to 2.302 mM (addition of *D*- and *L*-lactate concentrations). For the lactic acid condition, the final concentration of lactic acid was 22.302 mM. The concentration of lactic acid in TH broth was measured using enzymatic kits that measure *D*-lactate and *L*-lactate isomers (Eton Bioscience, San Diego, California).

### Metabolomics

After 48 h of incubation at 37°C, cell cultures from the 96-well plate were collected and centrifuged to collect supernatants. Approximately 20 μL of supernatant from each sample was immediately transferred to a new microcentrifuge tube and stored at –80°C. Supernatants were sent to the West Coast Metabolomics Center (WCMC) for untargeted metabolomics on a gas chromatography time of flight mass spectrometry (GCTOF-MS) platform. Metabolites were extracted with a mixture of 3:3:2 acetonitrile: isopropyl alcohol: water according to standard operating procedures from the Fiehn Lab at the WCMC (Cajka and Fiehn 2016).

### RNA sequencing

Cell pellets were resuspended in trizol and stored at –80°C immediately after centrifugation and removal of supernatant. Upon extraction, pellets were thawed on ice. Total RNA was extracted using the Direct-zol RNA extraction kit from Zymo, San Diego, California. We used Ribo-Zero specific for bacteria to remove ribosomal RNA (Illumina, San Diego, California). Libraries were built using the Illumina TruSeq Stranded mRNA library protocol. Libraries were sent to the UC Irvine sequencing core for 250 paired end sequencing on the HiSeq 2500.

### Phenotypic assays

Swarming and swimming phenotypic assays were performed. For the swarming assay, 5 μL of overnight culture was dropped onto a TH 0.35% agar plate and incubated at 37°C for 48 h. The swimming assay was performed on TH 0.3% agar. Plates with 2,3-butanediol and lactic acid were at 20 mM final concentration. Images of plates were taken with an Epson scanner. Colony area was quantified with ImageJ (https://imagej.net).

### Phylogenetic analysis

Phylogenetic analysis was performed on PaFLR01 and 19 CF isolates and common lab *P. aeruginosa* strains. The 19 additional *P. aeruginosa* genomes were downloaded from the NCBI Genbank repository (Table S2, Supporting Information). These strains were chosen based on deposited metadata indicating they were isolated from CF patients or are common lab strains, in addition to completeness of their genome assembly. Coding DNA sequences were extracted from each genome and protein BLAST alignments were performed (Camacho *et al.*^2009^) against the other genomes. Geneparser (https://github.com/mmmckay/geneparser) was used to parse the BLAST output, extract, and concatenate genes that are shared at 90% amino acid identity (AAI) across 90% of the protein sequence. These concatenated sequences were aligned using Multiple Alignment using Fast Fourier Transform (MAFFT) (Katoh *et al.*^2002^) using parameters –auto and –maxiterate 2. The phylogenetic tree was built with FastTree (Price, Dehal and Arkin 2010) using parameters –slow, –spr 4, –mlacc 2. Tree visualization was done using interactive Tree of Life (iTOL) (Letunic and Bork 2007).

### Transcriptome analysis

Quality control and filtering of transcriptome data were performed with Trimmomatic, PEAR (Paired-End reAd mergeR) and Deconseq. Trimmomatic trimmed adapter sequences, low-quality reads and removed Ns (Bolger, Lohse and Usadel 2014). PEAR combined overlapping paired reads into a single read (Zhang *et al.*^2014^). Deconseq removed remaining rRNA (Schmieder and Edwards 2011). Quality filtered reads were aligned to the reference PaFLR01 genome with Bowtie2 (Langmead and Salzberg 2012) and gene counts were generated using HTSeq (Anders, Pyl and Huber 2015). One of the biological replicates from the PaFLR01 control was removed from further downstream analyses due to low coverage (Table S3, Supporting Information). Differences in coverage were accounted for through normalization before the differential expression analysis, and normalized coverage across housekeeping genes and across the genome were as expected (Fig. S1a and b, Supporting Information). Differential expression gene analysis was performed with DESeq2, which contains an internal normalization step (Love, Huber and Anders 2014). The cutoff threshold for differentially expressed genes was a magnitude log2 fold change >1.5 and p value < 0.05. Gene categories from RAST were tested for differential expression by counting differentially expressed genes with a positive and negative log2 fold change and performing the binomial test in R to identify whether each category was differentially up- or down-regulated.

### Metabolome analysis

Raw data are provided in Table S4, Supporting Information. Metabolomics data analysis was performed in R and Primer (http://www.primer-e.com/Primary_papers.htm). In R, randomForest was performed with the randomForest package to identify metabolites that best distinguish each sample group or category from each other. Multivariate analysis including principle coordinate analysis (PCoA) was performed in primer. Metabolites and transcripts were normalized by total sum per sample.

## RESULTS

### Phylogenetic analysis of a clinical isolate and CF *P. aeruginosa* strains

**Figure 1.**
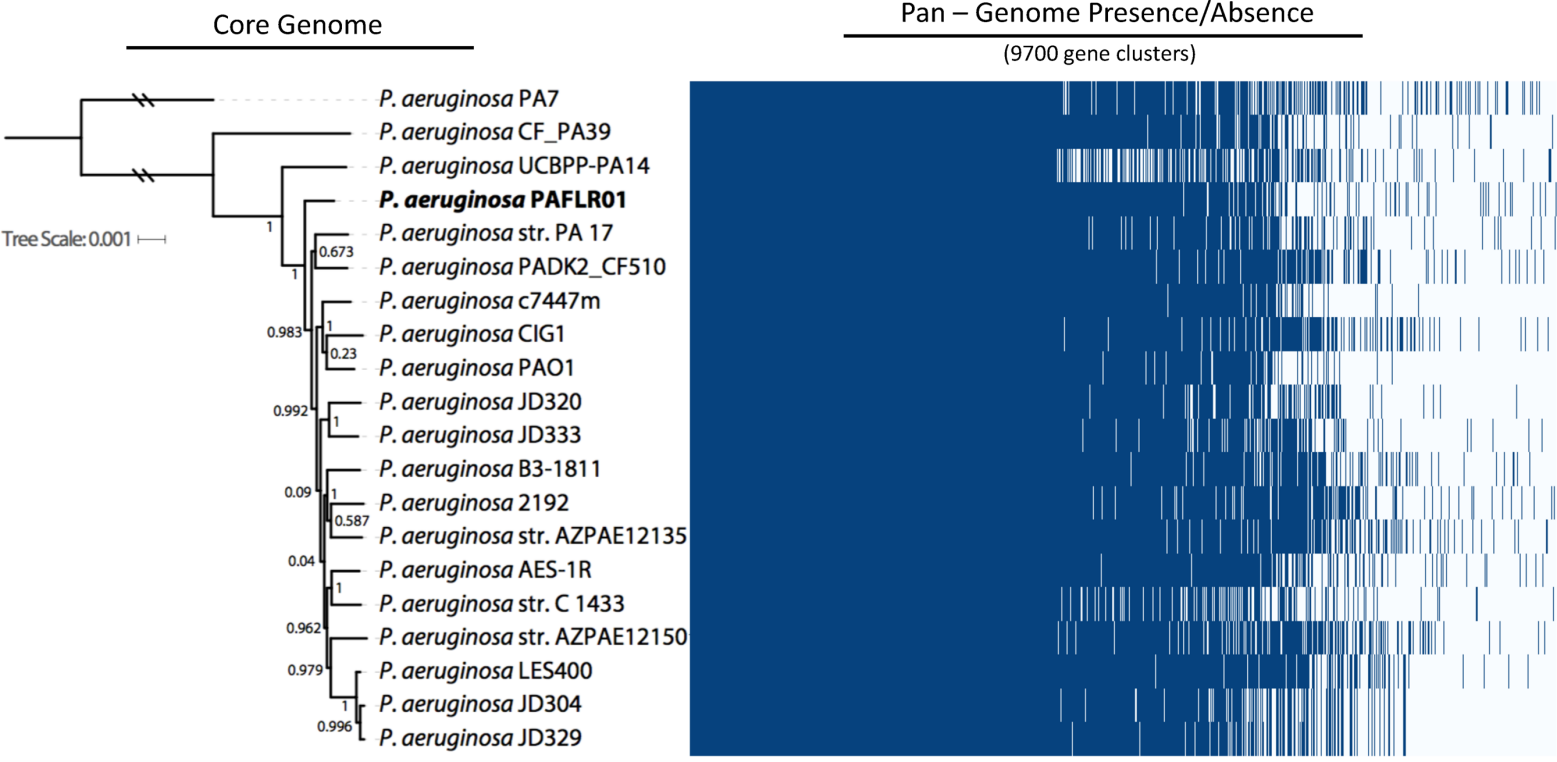
Phylogenetic tree and heatmap based on the core and pan-genome, respectively, of 20 *P. aeruginosa* common lab and CF isolates. One thousand three hundred sixty-nine genes are shared at 90% average AAI across 90% of the protein sequence. Values next to branches indicate bootstrap scores. Blue and white bars, respectively, represent presence and absence of genes across all 20 strains.

In order to identify the evolutionary and phylogenetic relationship between our clinical isolate and other common lab and CF *Pseudomonas aeruginosa* strains, we built a phylogenetic tree based on a core genome analysis and performed a BLAST search of the essential genome for *P. aeruginosa* in CF sputum. The three common lab strains included in this analysis were PA7, PAO1 and PA14. The other strains used in this study were CF clinical isolates, including the PaFLR01 clinical isolate used in this study. Core phylogenetic analysis indicates that PaFLR01 is closely related to many known CF isolates (Fig. 1). On average, the *P. aeruginosa* strains included in our analysis share 98.4% AAI across the core genome. However, the accessory genome of these *P. aeruginosa* strains contains many open reading frames that are not shared among all strains (Fig. 1). At 90% AAI, PaFLR01 contains 177 genes that are not shared with the other genomes used in this analysis.

When comparing PaFLR01 to PA14, a ubiquitously studied strain, we performed a BLAST search of the essential genome of PA14 in CF sputum (Turner *et al.*^2015^). The 508 genes determined to be essential for PA14 survival in CF and MOPS(3-(N-morpholino)propanesulfonic acid)-sputum (Turner *et al.*^2015^) were used as a BLAST reference for the 20 *P. aeruginosa* strains. PaFLR01 contains 493 of the 508 essential genes at an average protein identity of 99.7% for surviving in CF sputum (Table S1, Supporting Information). PaFLR01 contained more CF essential genes than 16 of the other genomes in the phylogenetic analysis.

### Phenotypic response of PaFLR01 to fermentation products

**Figure 2.**
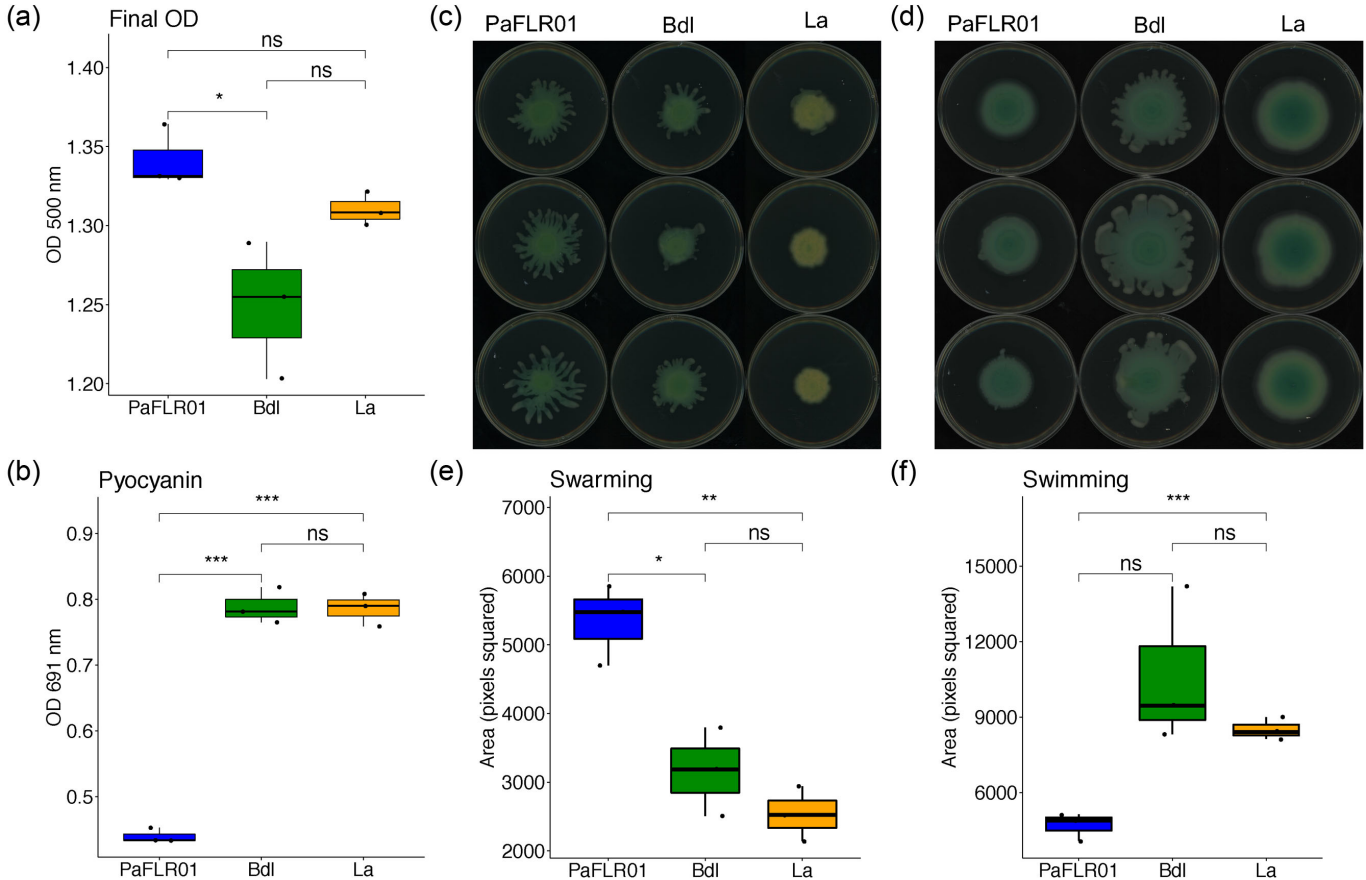
Phenotypic response of PaFLR01 to fermentation products. (**a**) Final optical density (OD) at 500 nm and (**b**) pyocyanin measurements at 691 nm were taken after 48 h of incubation at 37°C. Phenotypic (**c**) swarming and (**d**) swimming assays were set at 48 h at 37°C. Area quantification of (**e**) swarming and (**f**) swimming colonies was performed with ImageJ. Dots within each boxplot indicate each biological replicate. Bdl = PaFLR01 + 20 mM 2,3-butanediol. La = PaFLR01 + 20 mM lactic acid. Pairwise T tests were performed to determine significance. *p value < 0.05; **p value < 0.01; ***p value < 0.001; ns = p value > 0.05, not significant.

To investigate how a clinical isolate responds to fermentation products that are potential biomarkers of CF disease progression, we grew PaFLR01 in the presence of either 20 mM 2,3-butanediol or 20 mM lactic acid in biological triplicates for a period of 48 h at 37°C in a static 96-well plate. As a control, triplicates of PaFLR01 cultures were grown in culture without added metabolites. After 48 h, final optical density (OD; Fig. 2a) and pyocyanin concentrations (Fig. 2b) were recorded. Although the added metabolites did not significantly boost PaFLR01 growth, 2,3-butanediol and lactic acid nearly doubled the production of pyocyanin in comparison to the control (Fig. 2b).

In addition to pyocyanin measurements, we also looked at the coverage in counts per million across genes involved in phenazine production, specifically, *phzM* and *phzS* (Fig. S1c and d, Supporting Information). As a control, coverage of a housekeeping gene, DNA gyrase subunit B *gyrB* and coverage across the whole genome were examined (Fig. S1a and b, Supporting Information). *PhzM* is expressed at the same level in each of the 3 conditions, given even coverage across samples (Fig. S1c, Supporting Information). *PhzS* is up-regulated when exposed to 2,3-butanediol and lactic acid (Fig. S1d, Supporting Information).

Swarming and swimming assays were performed to investigate how PaFLR01 responds to lactic acid and 2,3-butanediol. Crystal violet biofilm assays were also performed, but provided inconsistent results due to lack of adherence to wells (data not shown). To keep conditions similar across experiments, we performed the swarming and swimming assays on TH plates. Swarming was significantly decreased when PaFLR01 was exposed to 2,3-butanediol and lactic acid (Fig. 2c and e). PaFLR01 control and lactic acid conditions display the swimming phenotype, but the 2,3-butanediol condition displays a swarming phenotype closer to the outer edge of the colony (Fig. 2d). This may be attributed to the additional carbon source available to PaFLR01 with the addition of 20 mM 2,3-butanediol.

### Metabolome profile of PaFLR01 growth in 2,3-butanediol and lactic acid

**Figure 3.**
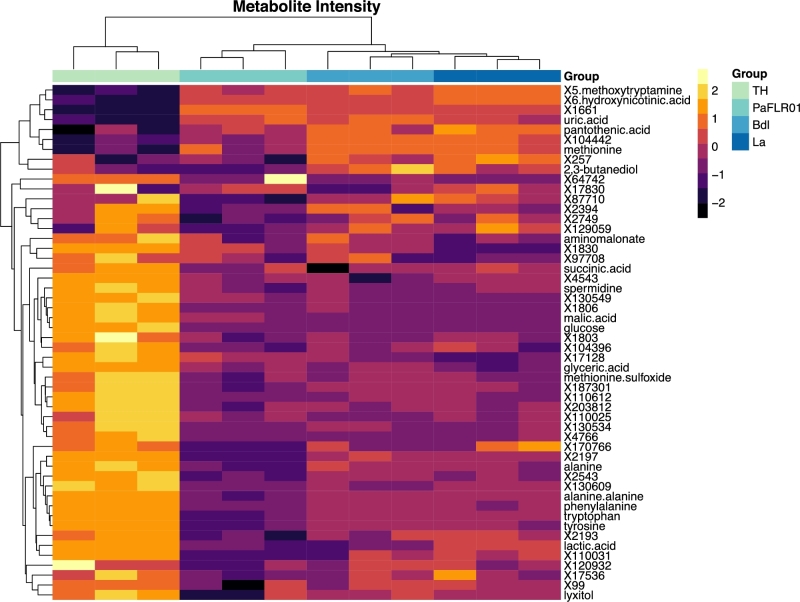
Heatmap of important metabolites chosen based on the randomForest algorithm. The top 50 metabolites output from the randomForest algorithm with the addition of glucose and 2,3-butanediol are presented. Metabolites were normalized by a log2 and scale transformation in R. The legend represents the z-scores, standard deviation from the mean, of the log2 transformed data. TH = Todd Hewitt media blank. La = PaFLR01 + 20 mM lactic acid. Bdl = PaFLR01 + 20 mM 2,3-butanediol. The metabolites labeled with an X and number are unannotated, although the ion can be consistently identified across samples. Dendrograms are hierarchically clustered based on Euclidean distances.

Metabolomes were measured by GCTOF-MS in an untargeted approach. The extracellular metabolomes of each growth condition formed distinct hierarchical clusters based on Euclidean distances (Fig. 3, top dendrogram). The two large clusters on the left y-axis dendrogram distinguish metabolites present at low and high abundances in the TH media blank that were produced and consumed by PaFLR01, respectively. Lactic acid is present at a concentration of 2.302 mM in the TH media and is consumed by PaFLR01 in all conditions (Fig. 3). Amino acids present in the TH media blank, alanine, tyrosine, tryptophan and phenylalanine, show decreased relative abundance in the PaFLR01 + 2,3-butanediol and PaFLR01 + lactic acid conditions compared to the TH control, indicating consumption or modification (Fig. 3). However, amino acids are even lower in the PaFLR01 control (Fig. 3).

### Differentially expressed gene categories of PaFLR01 growth in 2,3-butanediol and lactic acid

**Figure 4.**
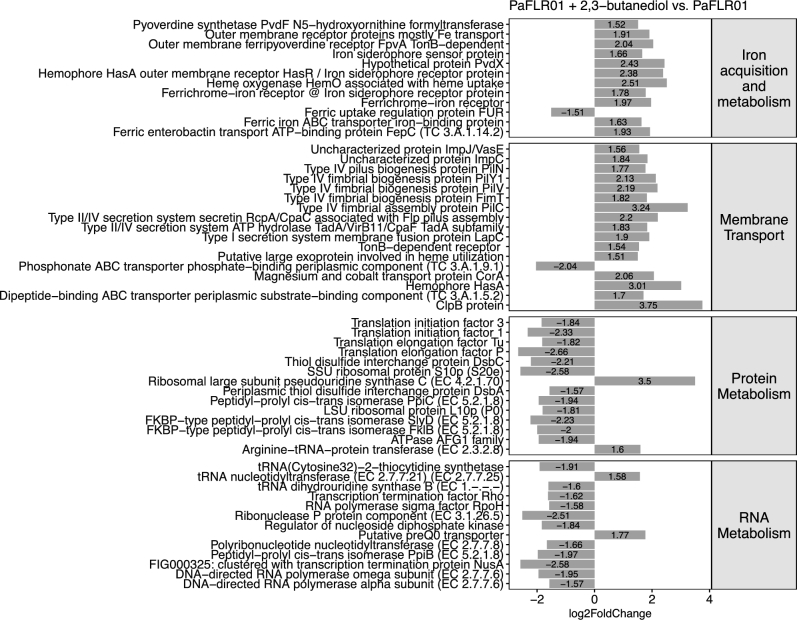
Differentially expressed SEED gene categories in PaFLR01 + 2,3-butanediol versus PaFLR01 control. Gene categories were selected based on the binomial test with a p value < 0.05. Genes within each category with a magnitude log2 fold change of >1.5 and p value of <0.05 are listed.

**Figure 5.**
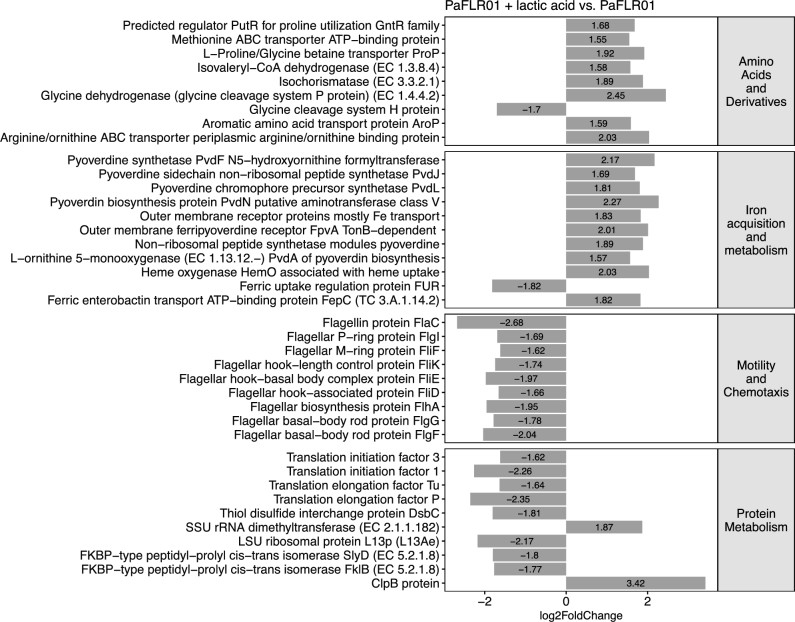
Differentially expressed SEED gene categories in PaFLR01 + lactic acid versus PaFLR01 control. Gene categories were selected based on the binomial test with a p value < 0.05. Genes within each category with a magnitude log2 fold change of >1.5 and p value of <0.05 are listed.

To identify SEED (http://www.theseed.org) cellular process categories that are significantly up- or down-regulated with a p value of <0.05, a binomial test was performed on the number of genes that had a p value of <0.05 and a magnitude log2 fold change > 1.5 in each SEED category. For PaFLR01 + 2,3-butanediol versus PaFLR01 control, iron acquisition metabolism and membrane transport gene categories are up-regulated while protein metabolism and RNA metabolism are down-regulated (Fig. 4). For PaFLR01 + lactic acid vs. PaFLR01 control, amino acids and derivatives and iron acquisition metabolism are up-regulated while motility and chemotaxis and protein metabolism are down-regulated (Fig. 5).

In iron acquisition and metabolism, pyoverdine synthetases (*pvdF* for PaFLR01 + 2,3-butanediol, and *pvdJ, pvdL* and *pvdA* for PaFLR01 + lactic acid) are up-regulated (Figs 4 and 5; iron acquisition metabolism). *FpvA*, a pyoverdine specific receptor that recognizes ferripyoverdine, is also up-regulated (Figs 4 and 5; iron acquisition metabolism). *FpvA* works in conjunction with *tonB*, up-regulated in PaFLR01 + 2,3-butanediol, a protein that is used to import ferripyoverdine into the periplasm of the cell (Schalk *et al.*^2001^; Adams *et al.*^2006^). For both 2,3-butanediol and lactic acid conditions, the *fur* repressor protein is down-regulated (Figs 4 and 5; iron acquisition metabolism). When ferrous iron is accumulated at high concentrations within the cell, *fur* represses the iron import system (Ochsner, Vasil and Vasil 1995; Hassett *et al.*^1996^).

Membrane transport is up-regulated in PaFLR01 + 2,3-butanediol, but not in PaFLR01 + lactic acid. In particular, type IV fimbrial biogenesis pilus genes (*pilN, pilY1, pilV, fimT* and *pilC*) are differentially up-regulated (Fig. 4, membrane transport). In the motility and chemotaxis gene category, flagellar genes involved in flagellum-mediated motility and adhesion (*flaC, flgL, fliF, fliK, fliE, fliD, flhA, flgG* and *flgF*) are differentially down-regulated in PaFLR01 + lactic acid but not in the PaFLR01 + 2,3-butanediol condition (Fig. 5, motility and chemotaxis).

### Relating metabolomes to transcriptomes

**Figure 6.**
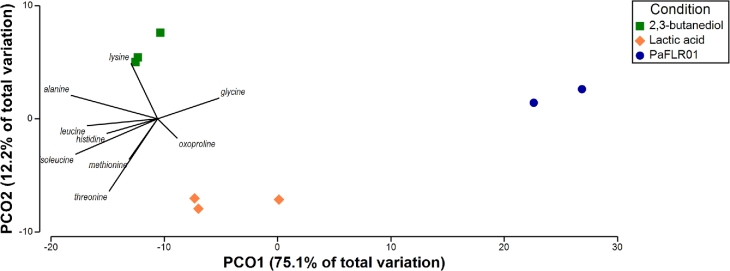
Principal coordinate analysis (PCoA) of transcriptomes overlaid with distinguishing metabolites. A PCoA with the Bray–Curtis similarity statistic was performed on the transcriptome dataset. Transcriptome counts were normalized by total number of gene counts per sample. Metabolites were normalized by total sum intensity per sample and were chosen based on best distinguishing each sample from each other. Metabolites included are threonine, oxoproline, methionine, lysine, leucine, isoleucine, hydroxylamine, histidine, glycine and alanine. 2,3-butanediol = PaFLR01 + 20 mM 2,3-butanediol. Lactic acid = PaFLR01 + 20 mM lactic acid.

To identify overlapping trends between the transcriptome and metabolome profiles, we performed a PCoA of the transcriptional data overlaid with metabolites selected based on best distinguishing each sample condition. The biological replicates for each condition of the transcriptome data clustered together (Fig. 6). A signature that arises is the correlation of amino acids with the PaFLR01 + lactic acid condition, also evident in the transcriptome data. For example, the predicted regulator *putR* for proline utilization is up-regulated; oxoproline is more closely clustered with the lactic acid condition in the overlaid PCoA (Fig. 6). In addition, amino acid transport proteins for methionine, *L*-proline, glycine, arginine, ornithine and aromatic acid transport protein *aroP* are also up-regulated (Fig. 5). Several of the amino acids associated with the genes listed above, specifically methionine, glycine and oxoproline are more associated with PaFLR01 exposure to fermentation products than to the PaFLR01 control (Fig. 6). The cluster of metabolites is centered between the PaFLR01 + 2,3-butanediol and PaFLR01 + lactic acid conditions, indicating that the amino acids are more associated with PaFLR01 exposure to fermentation products than the PaFLR01 control (Fig. 6).

## DISCUSSION

Understanding how pathogens like *Pseudomonas aeruginosa* respond to important metabolites from the CF lung may shed light on pathogen physiology *in vivo*. To work within a physiologically relevant range, we decided on 20 mM for both metabolites (further discussion and review of clinical sample measurements of 2,3-butanediol and lactic acid that motivated our experimental design in S1, Supporting Information). We also chose to focus on a late time point, 48 h after inoculation. Many biofilm studies focus on growth stages much earlier than what is reflected in an infection where most cells are likely slow-growing and well past stationary phase. Overall, we find that the addition of relevant concentrations of the fermentation products leads to changes in the aggregative or biofilm modes of growth, to use the terminology from DePas 2016 (DePas *et al.*^2016^), both in the physical handling properties of the clinical isolate PaFLR01 in the culturing experiments, and in the expression patterns from the transcriptomes.

### Fermentation products as a signal for low oxygen

Contrary to the conventional wisdom that lungs are well aerated and aerobic, oxygen does not penetrate liquid easily, and while hemoglobin carries oxygen to tissues that are accessible to circulating blood, dense mucus layers in the airways are largely inaccessible to fresh oxygenated hemoglobin. Neutrophils also consume much of the available oxygen (Kolpen *et al.*^2010^), limiting *P. aeruginosa* growth (Kragh *et al.*^2014^). Furthermore, fermentation products 2,3-butanediol and lactic acid may act as signals for low oxygen. In a transposon mutagenesis experiment, genes essential for survival in oxygen-limited conditions include transcriptional regulation and signal transduction, cell wall and phospholipid metabolism, transport, amino acid metabolism and proteolysis, among other gene categories (Basta, Bergkessel and Newman 2017). In our transcriptome data, we see an increase in expression in transport genes in the 2,3-butanediol condition and an increase in expression in amino acid metabolism genes in the lactic acid condition (Figs 4 and 5). The overlap in gene categories essential for oxygen limitation and up-regulated in response to fermentation products are consistent with the possibility that fermentation products act as a signal for low oxygen.

In low oxygen conditions, it has been shown that phenazine production by *Pseudomonas spp.* increases (van Rij *et al.*^2004^). In the pyocyanin production pathway, *phzM* converts phenazine-1-carboxylic acid to 5-methylphenazine-1-carboxylic acid betaine, which *phzS* converts to pyocyanin through the use of NADH and oxygen (Mavrodi *et al.*^2001^). Interestingly, while *phzS* is oxygen dependent and *phzM* is not, low oxygen signals from the fermentation products 2,3-butanediol and lactic acid induce an overexpression of *phzS*, but not *phzM* (Fig. S1c and d, Supporting Information). This result may reflect how fermentation products are signals of low oxygen and induce the production of phenazines that can act as alternative electron acceptors.

### Central role of iron acquisition and metabolism

Iron is required for respiration, biofilm formation and many other metabolic processes necessary for *P. aeruginosa* growth. Pyoverdine, one of the two major siderophores produced by *P. aeruginosa*, is critical for scavenging iron in a mouse lung infection model (Minandri *et al.*^2016^) and chronic biofilm infections (Turner *et al.*^2014^). Mutants unable to produce pyoverdine were shown to be deficient in biofilm formation. Increased expression of pyoverdine synthesis and receptor proteins and decreased expression of the *fur* repressor protein in our transcriptome data implies that cells are actively producing pyoverdine and importing ferrous iron (Figs 4 and 5). Therefore, the differential expression of these genes indicate that exposure to 2,3-butanediol and lactic acid induces change in PaFLR01 iron metabolism involved in respiration, biofilm formation or virulence.

### Aggregative growth and dormancy physiology in response to fermentation products

Several types of data support the idea that fermentation products push PaFLR01 toward aggregative growth and dormancy physiology. The evidence includes the increase in phenazine production described above, along with changes in expression of genes related to motility, protein metabolism and amino acid utilization described below.

Classic *in vitro* biofilm studies do not necessarily reflect the true physiology of microbes *in vivo* in CF lungs, but do, however, offer a glimpse into important biological features. For example, when *P. aeruginosa* cells attach to a surface to form a monolayer, genes involved in twitching motility and virulence are required for the initial stages of biofilm initiation (O’Toole and Kolter 1998; Comolli *et al.*^1999^). Bacteria living in the CF airways can be observed with a new tissue clearing technique called MiPACT (microbial identification after passive clarity technique), which enables detection of spatial distribution and aggregate size of clusters of bacterial cells in CF sputum (DePas *et al.*^2016^). Bacteria within sputum exist as single cells, medium-sized clusters and larger aggregates (DePas *et al.*^2016^). Furthermore, Sønderholm *et al.* recently showcased a new alginate bead model that leads to the growth of physiologically relevant aggregates of *P. aeruginosa* cells, advancing the field from traditionally studied surface attached biofilms (Sønderholm *et al.*^2018^).

Type IV pili are required for twitching motility and are important for surface adhesion and virulence (Bradley 1980; O’Toole and Kolter 1998; Comolli *et al.*^1999^; Persat *et al.*^2015^). In order to assemble a biomass of cells that form a biofilm, cells need to be able to sense and move towards one another. Pili genes necessary for twitching and swarming motility are up-regulated in the PaFLR01 + 2,3-butanediol condition. Results from the swarming assay show that 2,3-butanediol decreases swarming in PaFLR01 (Fig. 2d and f). In George O’Toole's model for biofilm formation, type IV pili may play an important role in early microcolony and biofilm formation (O’Toole and Kolter 1998). As also seen in *Clostridium difficile*, type IV pili are critical for early biofilm formation; *pilA* mutants are capable of forming biofilms but at a significantly reduced biomass (Maldarelli *et al.*^2016^). *Pil* gene mutations are also a common adaptation of *Pseudomonas* in the CF lung (Winstanley, O’Brien and Brockhurst 2016).

Once a *P. aeruginosa* biofilm is established, the majority of cells within the biofilm are no longer motile (Caiazza *et al.*^2007^; Kuchma *et al.*^2007^; Merritt *et al.*^2007^). In the swarming assay, when PaFLR01 is exposed to lactic acid, swarming significantly decreases (Fig. 2c and e). Genes involved in motility, including *pil* genes, *rpoN* and *cup* genes are often mutated in adaptation to the CF lung (Winstanley, O’Brien and Brockhurst 2016). *Pseudomonas* also adapts to the CF environment by repressing flagellar genes to avoid eliciting host defenses (Wolfgang *et al.*^2004^). When PaFLR01 is exposed to lactic acid, flagella genes are significantly down-regulated (Fig. 5). Although PaFLR01 uses lactic acid as a carbon source, forming a biofilm in response to exposure to lactic acid protects itself against acidity, a signal for low oxygen. In addition to the defense of a biofilm, a low-pH environment also inhibits antimicrobial susceptibility. In a porcine CF model, decrease in pH inhibited the antimicrobial activity of airway surface liquid against bacterial killing (Pezzulo *et al.*^2012^). The up-regulation of genes involved in biofilm formation likely provides a survival advantage to *P. aeruginosa* in the acidic lung environment of CF patients.

### Reduction in protein metabolism

Cells in a biofilm are more sessile and dormant than planktonic cells. Addition of 2,3-butanediol and lactic acid led to a decrease in expression of genes involved in protein metabolism (Figs 4 and 5). 2,3-butanediol also led to a decrease in RNA metabolism (Fig. 4). This further supports our hypothesis that exposure to lactic acid and 2,3-butanediol alter physiology in clinically relevant ways. In particular, a down-regulated gene *slyD*, a molecular chaperone, is a key molecule involved in cell growth (Kumar and Balbach 2017). Additionally, *fklB*, a gene that has been shown to increase susceptibility of *P. aeruginosa* to B-lactams when mutated is also down-regulated (Alvarez-Ortega *et al.*^2010^). As *P. aeruginosa* starts to decrease growth, they may become more physiologically similar to conditions *in vivo*, as *P. aeruginosa* is more slowly growing and dormant (Babin *et al.*^2016^). When in a biofilm, *P. aeruginosa* does not need to express *flkB* to defend itself against antibiotics because the biofilm and state of growth will protect *P. aeruginosa* from several classes of antibiotics.

### Amino acid utilization

In the present study, both expression data and metabolites show increased PaFLR01 amino acid consumption with exposure to fermentation products (Figs 3 and 5). As *P. aeruginosa* strains adapt to the CF environment over the course of years, genes and metabolites involved in amino acid metabolism tend to increase (Hoboth *et al.*^2009^; Behrends *et al.*^2013^). In a study of 179 CF adapted *P. aeruginosa* isolates, Behrends *et al.* showed that CF adaptations included increased efficiency in utilizing amino acids, likely because of their abundance in CF sputum and because amino acids are metabolically expensive (Behrends *et al.*^2013^). However, there was not a convergent metabolism for all *P. aeruginosa* strains surveyed *in vitro* because of the different selection pressures for evolution and adaptation in each patient (Behrends *et al.*^2013^).

### Unknowns

Less than half of the metabolites detected by untargeted GCTOF-MS are known because of incomplete libraries and identification methods (227 of 706 are annotated in this study). A typical experiment with clinical samples yields thousands or tens of thousands of metabolites. From LC-MS (Liquid Chromatography Mass Spectrometry) data often less than 2% can be identified (da Silva, Dorrestein and Quinn 2015), while GC-MS databases are better developed and the molecules are often better-studied central metabolites, so closer to half of them are often identified, as we see in this dataset.

## CONCLUSION

Volatile molecules have wide range and can impact the physiology of even distantly located pathogens. Whether 2,3-butanedione producing *Streptococcus* spp and *Rothia mucilaginosa* (Whiteson *et al.*^2014^; Phan *et al.*^2017^) occupy the oral cavity or another part of the airways, the fermentation products they produce may reach opportunistic pathogens such as *Pseudomonas aeruginosa*. Fermentation products may be a nutrient or a signal of low oxygen conditions, and induce chronic infection associated physiology including phenazine production, bacterial cell aggregation and dormancy.

## SUPPLEMENTARY DATA

Supplementary data are available at *FEMSLE* online.

## Supplementary Material

Supplementary Data
