# Supplementary material for: Fermentation products in the cystic fibrosis airways induce aggregation and dormancy-associated expression profiles in a CF clinical isolate of Pseudomonas aeruginosa
Source: FEMS Microbiol Lett. 2018 Mar 29;365(10):fny082. doi: 10.1093/femsle/fny082 (PMC5928460; doi:10.1093/femsle/fny082)
Supplement: Supplementary Data [file fny082_supp.zip › SupplementaryFigure1_revision2.pdf]

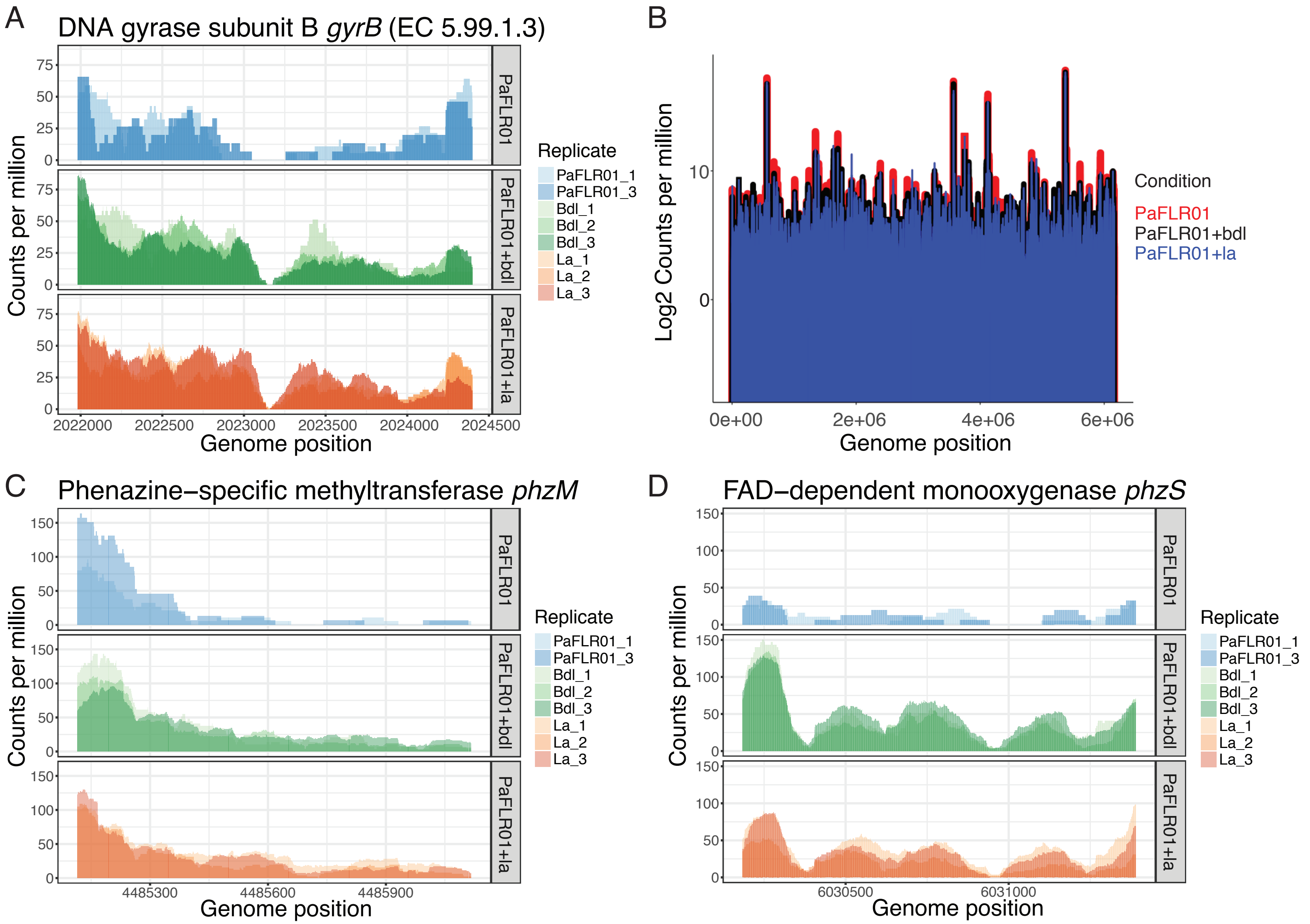

A
DNA gyrase subunit B gyrB (EC 5.99.1.3)
Counts per million
75
50
25
0
PaFLR01
PaFLR01+bdl
PaFLR01+la
2022000
2022500
2023000
2023500
2024000
2024500
Genome position
Replicate
PaFLR01_1
PaFLR01_3
Bdl_1
Bdl_2
Bdl_3
La_1
La_2
La_3
B
Log2 Counts per million
10
0
0e+00
2e+06
4e+06
6e+06
Genome position
Condition
PaFLR01
PaFLR01+bdl
PaFLR01+la
C
Phenazine−specific methyltransferase phzM
Counts per million
150
100
50
0
4485300
4485600
4485900
Genome position
D
FAD−dependent monooxygenase phzS
6030500
6031000
