# Supplementary material for: Fermentation products in the cystic fibrosis airways induce aggregation and dormancy-associated expression profiles in a CF clinical isolate of Pseudomonas aeruginosa
Source: FEMS Microbiol Lett. 2018 Mar 29;365(10):fny082. doi: 10.1093/femsle/fny082 (PMC5928460; doi:10.1093/femsle/fny082)
Supplement: Supplementary Data [file fny082_supp.zip › Supplementary_material_S1.docx]

Supplementary material S1

The concentration of metabolites chosen for this study was based on *in vitro* titration growth curves (Supplementary Figure 2) and data found in literature. In CF sputum, lactate concentrations are reported to range from 450 uM up to 15 mM (Bensel et al., 2011; Palmer et al., 2007; Twomey et al., 2013). Palmer et al. developed a synthetic CF sputum medium (SCFM) in which they quantified the concentrations of components of CF sputum and determined that lactate concentration was at 9 mM, but the expectorated sputum came from 12 stable individuals with CF (Palmer et al., 2007). In Twomey et al., lactate concentrations from 26 sputum samples from patients experiencing an exacerbation event ranged from 200 uM up to approximately 600 uM (Twomey et al., 2013). In Bensel et al., lactate was measured in 18 exacerbated and 25 stable CF patients and was found to be around 3.4 mmol/L compared to 1.4 mmol/L, respectively (Bensel et al., 2011). Differences in the concentration ranges between these studies may be due to technical or individual differences between patients. In our *in vitro* work, concentrations of lactic acid greater than 20 mM lactic acid inhibited PaFLR01 growth (Supplementary Figure 2B), while 20 mM lactic acid led to an increase in final optical density (Supplementary Figure 2B) and pyocyanin production (data not shown) compared to lower concentrations.

While there is no published work on the levels of 2,3-butanediol in CF sputum, previous work by another group found that *P. aeruginosa* exhibited increased growth and pyocyanin production when fed 30 mM 2,3-butanediol (Venkataraman et al., 2014). In an *in vivo* agar bead model, *P. aeruginosa* exposed to 30 mM 2,3-butanediol persisted for a longer period of time and increased the murine inflammatory response (Nguyen et al., 2016). In healthy individuals, 2,3-butanedione was detected at concentrations that ranged from 1.4-187 parts per billion (ppb) (Mochalski et al., 2013). Within the CF airways and sputum, there are steep gradients of metabolites such as lactic acid and 2,3-butanediol that depend on local production, consumption, and mixing.

Bensel, T., Stotz, M., Borneff-Lipp, M., Wollschläger, B., Wienke, A., Taccetti, G., Campana, S., Meyer, K.C., Jensen, P.Ø., Lechner, U., Ulrich, M., Döring, G., Worlitzsch, D., 2011. Lactate in cystic fibrosis sputum. J. Cyst. Fibros. Off. J. Eur. Cyst. Fibros. Soc. 10, 37–44. https://doi.org/10.1016/j.jcf.2010.09.004

Mochalski, P., King, J., Klieber, M., Unterkofler, K., Hinterhuber, H., Baumann, M., Amann, A., 2013. Blood and breath levels of selected volatile organic compounds in healthy volunteers. The Analyst 138, 2134–2145. https://doi.org/10.1039/c3an36756h

Nguyen, M., Sharma, A., Wu, W., Gomi, R., Sung, B., Hospodsky, D., Angenent, L.T., Worgall, S., 2016. The fermentation product 2,3-butanediol alters P. aeruginosa clearance, cytokine response and the lung microbiome. ISME J. https://doi.org/10.1038/ismej.2016.76

Palmer, K.L., Aye, L.M., Whiteley, M., 2007. Nutritional Cues Control Pseudomonas aeruginosa Multicellular Behavior in Cystic Fibrosis Sputum. J. Bacteriol. 189, 8079–8087. https://doi.org/10.1128/JB.01138-07

Twomey, K.B., Alston, M., An, S.-Q., O’Connell, O.J., McCarthy, Y., Swarbreck, D., Febrer, M., Dow, J.M., Plant, B.J., Ryan, R.P., 2013. Microbiota and Metabolite Profiling Reveal Specific Alterations in Bacterial Community Structure and Environment in the Cystic Fibrosis Airway during Exacerbation. PLOS ONE 8, e82432. https://doi.org/10.1371/journal.pone.0082432

Venkataraman, A., Rosenbaum, M.A., Werner, J.J., Winans, S.C., Angenent, L.T., 2014. Metabolite transfer with the fermentation product 2,3-butanediol enhances virulence by Pseudomonas aeruginosa. ISME J. 8, 1210–1220. https://doi.org/10.1038/ismej.2013.232
