# Supplementary figures and images for: Fermentation products in the cystic fibrosis airways induce aggregation and dormancy-associated expression profiles in a CF clinical isolate of Pseudomonas aeruginosa
Source: FEMS Microbiol Lett. 2018 Mar 29;365(10):fny082. doi: 10.1093/femsle/fny082 (PMC5928460; doi:10.1093/femsle/fny082)

Supplementary Figure 2

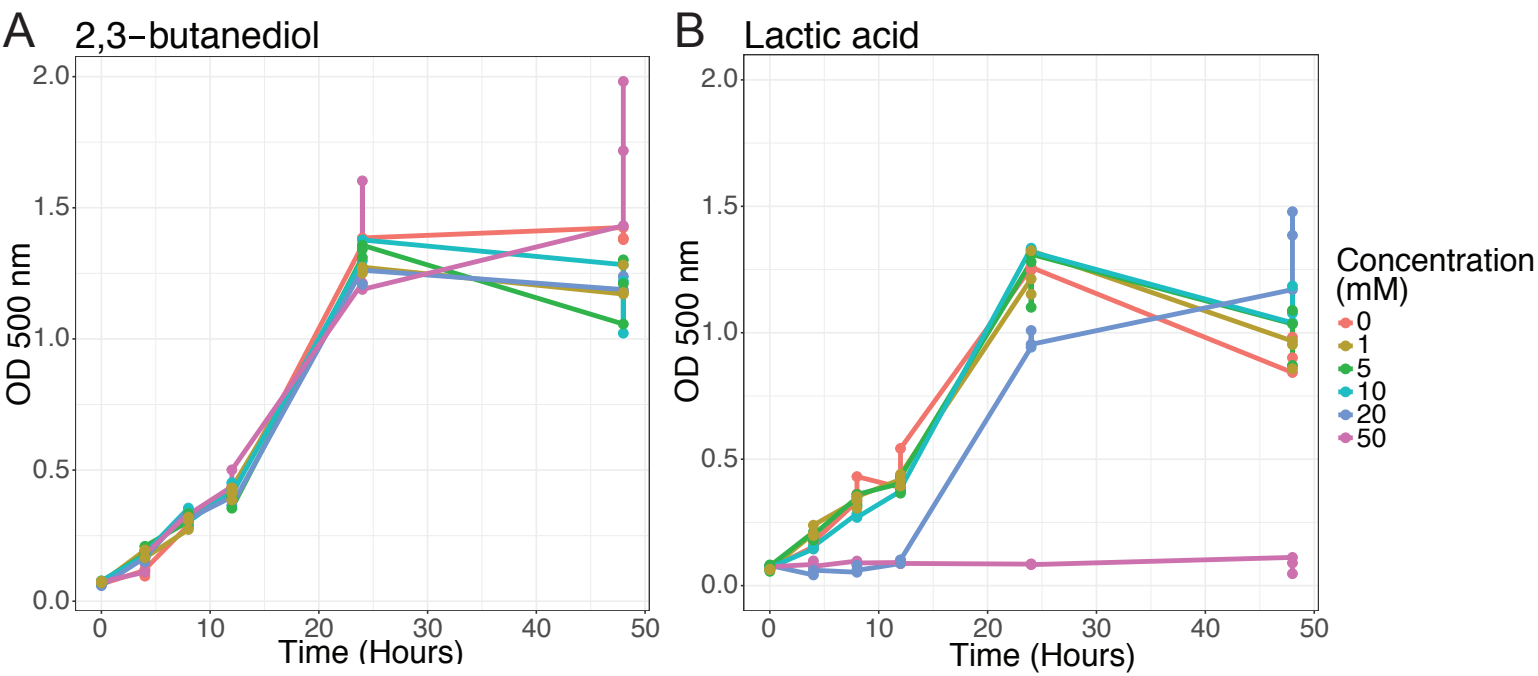

Supplement: Supplementary Data [file fny082_supp.zip › SupplementaryFigure2.pdf]
